# Antioxidant and free radical scavenging activity of *Spondias pinnata*

**DOI:** 10.1186/1472-6882-8-63

**Published:** 2008-12-09

**Authors:** Bibhabasu Hazra, Santanu Biswas, Nripendranath Mandal

**Affiliations:** 1 Division of Molecular Medicine, Bose Institute, P-1/12 CIT Scheme VIIM, Kolkata-700054, India

## Abstract

**Background:**

Many diseases are associated with oxidative stress caused by free radicals. Current research is directed towards finding naturally-occurring antioxidants of plant origin. The aim of the present study was to evaluate the *in vitro *antioxidant activities of *Spondias pinnata *stem bark extract.

**Methods:**

A 70% methanol extract of *Spondias pinnata *stem bark was studied *in vitro *for total antioxidant activity, for scavenging of hydroxyl radicals, superoxide anions, nitric oxide, hydrogen peroxide, peroxynitrite, singlet oxygen and hypochlorous acid, and for iron chelating capacity, reducing power, and phenolic and flavonoid contents.

**Results:**

The extract showed total antioxidant activity with a trolox equivalent antioxidant concentration (TEAC) value of 0.78 ± 0.02. The IC_50 _values for scavenging of free radicals were 112.18 ± 3.27 μg/ml, 13.46 ± 0.66 μg/ml and 24.48 ± 2.31 μg/ml for hydroxyl, superoxide and nitric oxide, respectively. The IC_50 _for hydrogen peroxide scavenging was 44.74 ± 25.61 mg/ml. For the peroxynitrite, singlet oxygen and hypochlorous acid scavenging activities the IC_50 _values were 716.32 ± 32.25 μg/ml, 58.07 ± 5.36 μg/ml and 127.99 ± 6.26 μg/ml, respectively. The extract was found to be a potent iron chelator with IC_50 _= 66.54 ± 0.84 μg/ml. The reducing power was increased with increasing amounts of extract. The plant extract (100 mg) yielded 91.47 ± 0.004 mg/ml gallic acid-equivalent phenolic content and 350.5 ± 0.004 mg/ml quercetin-equivalent flavonoid content.

**Conclusion:**

The present study provides evidence that a 70% methanol extract of *Spondias pinnata *stem bark is a potential source of natural antioxidants.

## Background

It is increasingly being realized that many of today's diseases are due to the "oxidative stress" that results from an imbalance between formation and neutralization of pro-oxidants. Oxidative stress is initiated by free radicals, which seek stability through electron pairing with biological macromolecules such as proteins, lipids and DNA in healthy human cells and cause protein and DNA damage along with lipid peroxidation. These changes contribute to cancer, atherosclerosis, cardiovascular diseases, ageing and inflammatory diseases [1,2]. All human cells protect themselves against free radical damage by enzymes such as superoxide dismutase (SOD) and catalase, or compounds such as ascorbic acid, tocopherol and glutathione [3]. Sometimes these protective mechanisms are disrupted by various pathological processes, and antioxidant supplements are vital to combat oxidative damage. Recently, much attention has been directed towards the development of ethnomedicines with strong antioxidant properties but low cytotoxicities.

*Spondias pinnata *(Linn. f.) Kurz (Family – Anacardiaceae) is a deciduous tree distributed in India, Sri Lanka and South-East Asian countries. In India it is commonly seen in the deciduous to semi-evergreen forests of the Western Ghats. The genus *Spondias *includes 17 described species, 7 of which are native to the neotropics and about 10 are native to tropical Asia. The phytochemistry of this plant has been studied [4]. The gum exudate of the species has been found to contain acidic polysaccharides [5]. A crude extract of *S. pinnata *has been reported to show antibacterial activity [6]. In ethnomedicine, equal quantities of bark juice of *S. pinnata *and *Syzygium cumuni *are prescribed as a remedy for dysentery [7]. An aqueous extract of this plant inhibits the citrus canker of lime [8]. However, there has been no report on the antioxidant properties of this species.

The objective of the present study was to evaluate the antioxidant potential and free radical scavenging activity of a 70% methanol extract of *S. pinnata*. The extract was examined for different reactive oxygen species (ROS) scavenging activities including hydroxyl, superoxide, nitric oxide, hydrogen peroxide, peroxynitrite, singlet oxygen and hypochlorous acid, and for phenol and flavonoid contents, iron chelating capacity and total antioxidant activity.

## Methods

### Chemicals

2,2'-azinobis-(3-ethylbenzothiazoline-6-sulfonic acid) (ABTS) was obtained from Roche Diagnostics, Mannheim, Germany. 6-hydroxy-2,5,7,8-tetramethychroman-2-carboxylic acid (Trolox) was obtained from Fluka, Buchs, Switzerland. Potassium persulfate (K_2_S_2_O_8_), ethylenediamine tetraacetic acid (EDTA), ascorbic acid, 2-deoxy-2-ribose, trichloroacetic acid (TCA), mannitol, nitro blue tetrazolium (NBT), reduced nicotinamide adenine dinucleotide (NADH), phenazine methosulfate (PMS), sodium nitroprusside (SNP), sulfanilamide, naphthylethylenediamine dihydrochloride (NED), L-histidine, lipoic acid, sodium pyruvate, quercetin and ferrozine were obtained from Sisco Research Laboratories Pvt. Ltd, Mumbai, India. Hydrogen peroxide, potassium hexacyanoferrate, Folin-Ciocalteu reagent, sodium carbonate (Na_2_CO_3_), butylated hydroxytoluene (BHT), sodium hypochlorite (NaOCl), aluminium chloride (AlCl_3_), ammonium iron (II) sulfate hexahydrate ((NH_4_)_2_Fe(SO_4_)_2_6H_2_O), potassium nitrite (KNO_2_), *N*, *N*-dimethyl-4-nitrosoaniline and xylenol orange were obtained from Merck, Mumbai, India. Gallic acid and curcumin were obtained from MP Biomedicals, France. Ferrous sulfate and catalase were obtained from HiMedia Laboratories Pvt. Ltd, Mumbai, India. Evans Blue was purchased from BDH, England. Manganese dioxide was obtained from SD Fine Chemicals, Mumbai, India. Diethylene-triamine-pentaacetic acid (DTPA) was obtained from Spectrochem Pvt. Ltd, Mumbai, India. Thiobarbituric acid (TBA) was obtained from Loba Chemie, Mumbai, India. Sodium nitrite was obtained from Qualigens Fine Chemicals, Mumbai, India.

### Plant material

The bark of the *S. pinnata *plant was collected from the Bankura district of West Bengal, India and authenticated through the Central Research Institute of Ayurveda, Kolkata, India.

### Extraction

The stem bark of *S. pinnata *was dried at room temperature for 7 days, finely powdered and used for extraction. The powder (100 g) was mixed with 500 ml methanol:water (7:3) using a magnetic stirrer for 15 hours, then the mixture was centrifuged at 2850 × *g *and the supernatant was decanted. The pellet was mixed again with 500 ml methanol-water and the entire extraction process was repeated. The supernatants collected from the two phases were mixed in a round bottom flask and concentrated under reduced pressure in a rotary evaporator. The concentrated extract was then lyophilized. The residue was kept at -20°C for future use.

### Total antioxidant activity

The ability of the test sample to scavenge ABTS^.+ ^radical cation was compared to trolox standard [9]. The ABTS^.+ ^radical cation was pregenerated by mixing 7 mM ABTS stock solution with 2.45 mM potassium persulfate (final concentration) and incubating for 12–16 h in the dark at room temperature until the reaction was complete and the absorbance was stable. The absorbance of the ABTS^.+ ^solution was equilibrated to 0.70 (± 0.02) by diluting with water at room temperature, then 1 ml was mixed with 10 μl of the test sample (0.05–10 mg/ml) and the absorbance was measured at 734 nm after 6 min. All experiments were repeated six times. The percentage inhibition of absorbance was calculated and plotted as a function of the concentration of standard and sample to determine the trolox equivalent antioxidant concentration (TEAC). To calculate the TEAC, the gradient of the plot for the sample was divided by the gradient of the plot for trolox.

### Hydroxyl radical scavenging

This was assayed as described by Elizabeth and Rao [10] with a slight modification. The assay is based on quantification of the degradation product of 2-deoxyribose by condensation with TBA. Hydroxyl radical was generated by the Fe^3+^-ascorbate-EDTA-H_2_O_2 _system (the Fenton reaction). The reaction mixture contained, in a final volume of 1 ml, 2-deoxy-2-ribose (2.8 mM); KH_2_PO_4_-KOH buffer (20 mM, pH 7.4); FeCl_3 _(100 μM); EDTA (100 μM); H_2_O_2 _(1.0 mM); ascorbic acid (100 μM) and various concentrations (0–200 μg/ml) of the test sample or reference compound. After incubation for 1 h at 37°C, 0.5 ml of the reaction mixture was added to 1 ml 2.8% TCA, then 1 ml 1% aqueous TBA was added and the mixture was incubated at 90°C for 15 min to develop the color. After cooling, the absorbance was measured at 532 nm against an appropriate blank solution. All tests were performed six times. Mannitol, a classical OH^. ^scavenger, was used as a positive control. Percentage inhibition was evaluated by comparing the test and blank solutions.

### Superoxide radical scavenging

This activity was measured by the reduction of NBT according to a previously reported method [11]. The non-enzymatic phenazine methosulfate-nicotinamide adenine dinucleotide (PMS/NADH) system generates superoxide radicals, which reduce nitro blue tetrazolium (NBT) to a purple formazan. The 1 ml reaction mixture contained phosphate buffer (20 mM, pH 7.4), NADH (73 μM), NBT (50 μM), PMS (15 μM) and various concentrations (0–20 μg/ml) of sample solution. After incubation for 5 min at ambient temperature, the absorbance at 562 nm was measured against an appropriate blank to determine the quantity of formazan generated. All tests were performed six times. Quercetin was used as positive control.

### Nitric oxide radical scavenging

At physiological pH, nitric oxide generated from aqueous sodium nitroprusside (SNP) solution interacts with oxygen to produce nitrite ions, which may be quantified by the Griess Illosvoy reaction [12]. The reaction mixture contained 10 mM SNP, phosphate buffered saline (pH 7.4) and various doses (0–70 μg/ml) of the test solution in a final volume of 3 ml. After incubation for 150 min at 25°C, 1 ml sulfanilamide (0.33% in 20% glacial acetic acid) was added to 0.5 ml of the incubated solution and allowed to stand for 5 min. Then 1 ml of napthylethylenediamine dihydrochloride (NED) (0.1% w/v) was added and the mixture was incubated for 30 min at 25°C. The pink chromophore generated during diazotization of nitrite ions with sulphanilamide and subsequent coupling with NED was measured spectrophotometrically at 540 nm against a blank sample. All tests were performed six times. Curcumin was used as a standard.

### Hydrogen peroxide scavenging

This activity was determined according to a previously described method [13] with minor changes. An aliquot of 50 mM H_2_O_2 _and various concentrations (0–2 mg/ml) of samples were mixed (1:1 v/v) and incubated for 30 min at room temperature. After incubation, 90 μl of the H_2_O_2_-sample solution was mixed with 10 μl HPLC-grade methanol and 0.9 ml FOX reagent was added (prepared in advance by mixing 9 volumes of 4.4 mM BHT in HPLC-grade methanol with 1 volume of 1 mM xylenol orange and 2.56 mM ammonium ferrous sulfate in 0.25 M H_2_SO_4_). The reaction mixture was then vortexed and incubated at room temperature for 30 min. The absorbance of the ferric-xylenol orange complex was measured at 560 nm. All tests were carried out six times and sodium pyruvate was used as the reference compound [14].

### Peroxynitrite scavenging

Peroxynitrite (ONOO^-^) was synthesized by the method described by Beckman et al. [15]. An acidic solution (0.6 M HCl) of 5 ml H_2_O_2 _(0.7 M) was mixed with 5 ml 0.6 M KNO_2 _on an ice bath for 1 s and 5 ml of ice-cold 1.2 M NaOH was added. Excess H_2_O_2 _was removed by treatment with granular MnO_2 _prewashed with 1.2 M NaOH and the reaction mixture was left overnight at -20°C. Peroxynitrite solution was collected from the top of the frozen mixture and the concentration was measured spectrophotometrically at 302 nm (ε = 1670 M^-1 ^cm^-1^).

An Evans Blue bleaching assay was used to measure peroxynitrite scavenging activity. The assay was performed by a standard method [16] with a slight modification. The reaction mixture contained 50 mM phosphate buffer (pH 7.4), 0.1 mM DTPA, 90 mM NaCl, 5 mM KCl, 12.5 μM Evans Blue, various doses of plant extract (0–200 μg/ml) and 1 mM peroxynitrite in a final volume of 1 ml. After incubation at 25°C for 30 min the absorbance was measured at 611 nm. The percentage scavenging of ONOO^- ^was calculated by comparing the results of the test and blank samples. All tests were performed six times. Gallic acid was used as the reference compound.

### Singlet oxygen scavenging

The production of singlet oxygen (^1^O_2_) was determined by monitoring *N*, *N*-dimethyl-4-nitrosoaniline (RNO) bleaching, using a previously reported spectrophotometric method [17,18]. Singlet oxygen was generated by a reaction between NaOCl and H_2_O_2_, and the bleaching of RNO was monitored at 440 nm. The reaction mixture contained 45 mM phosphate buffer (pH 7.1), 50 mM NaOCl, 50 mM H_2_O_2_, 50 mM histidine, 10 μM RNO and various concentrations (0–200 μg/ml) of sample in a final volume of 2 ml. It was incubated at 30°C for 40 min and the decrease in RNO absorbance was measured at 440 nm. The scavenging activity of sample was compared with that of lipoic acid, used as a reference compound. All tests were performed six times.

### Hypochlorous acid scavenging

Hypochlorous acid (HOCl) was prepared immediately before the experiment by adjusting the pH of a 10% (v/v) solution of NaOCl to 6.2 with 0.6 M H_2_SO_4_, and the concentration of HOCl was determined by measuring the absorbance at 235 nm using the molar extinction coefficient of 100 M^-1 ^cm^-1^. The assay was carried out as described by Aruoma and Halliwell [19] with minor changes. The scavenging activity was evaluated by measuring the decrease in absorbance of catalase at 404 nm. The reaction mixture contained, in a final volume of 1 ml, 50 mM phosphate buffer (pH 6.8), catalase (7.2 μM), HOCl (8.4 mM) and increasing concentrations (0–100 μg/ml) of plant extract. The assay mixture was incubated at 25°C for 20 min and the absorbance was measured against an appropriate blank. All tests were performed six times. Ascorbic acid, a potent HOCl scavenger, was used as a reference [20].

### Fe^2+ ^chelation

The ferrous ion chelating activity was evaluated by a standard method [21] with minor changes. The reaction was carried out in HEPES buffer (20 mM, pH 7.2). Briefly, various concentrations (0–120 μg/ml) of plant extract were added to 12.5 μM ferrous sulfate solution and the reaction was initiated by the addition of ferrozine (75 μM). The mixture was shaken vigorously and incubated for 20 min at room temperature, then the absorbance was measured at 562 nm. All tests were performed six times. EDTA was used as a positive control.

### Reducing power

The Fe^3+^-reducing power of the extract was determined by the method of Oyaizu [22] with a slight modification. Different concentrations (0.0–0.4 mg/ml) of the extract (0.5 ml) were mixed with 0.5 ml phosphate buffer (0.2 M, pH 6.6) and 0.5 ml potassium hexacyanoferrate (0.1%), followed by incubation at 50°C in a water bath for 20 min. After incubation, 0.5 ml of TCA (10%) was added to terminate the reaction. The upper portion of the solution (1 ml) was mixed with 1 ml distilled water, and 0.1 ml FeCl_3 _solution (0.01%) was added. The reaction mixture was left for 10 min at room temperature and the absorbance was measured at 700 nm against an appropriate blank solution. All tests were performed six times. A higher absorbance of the reaction mixture indicated greater reducing power. Butylated hydroxytoluene (BHT) was used as a positive control.

### Determination of total phenolic content

Total phenolic content was determined using Folin-Ciocalteu (FC) reagent according to the method of Singleton and Rossi [23] with a slight modification. Briefly, the plant extract (0.1 ml) was mixed with 0.75 ml of FC reagent (previously diluted 1000-fold with distilled water) and incubated for 5 min at 22°C, then 0.06% Na_2_CO_3 _solution was added. After incubation at 22°C for 90 min, the absorbance was measured at 725 nm. All tests were performed six times. The phenolic content was evaluated from a gallic acid standard curve.

### Determination of total flavonoid content

The total flavonoid content was determined with aluminium chloride (AlCl_3_) according to a known method [24] using quercetin as a standard. The plant extract (0.1 ml) was added to 0.3 ml distilled water followed by NaNO_2_ (0.03 ml, 5%). After 5 min at 25°C, AlCl_3 _(0.03 ml, 10%) was added. After a further 5 min, the reaction mixture was treated with 0.2 ml 1 mM NaOH. Finally, the reaction mixture was diluted to 1 ml with water and the absorbance was measured at 510 nm. All tests were performed six times. The flavonoid content was calculated from a quercetin standard curve.

### Statistical analysis

All data are given as the mean ± SD of six measurements. Statistical analysis was performed using KyPlot version 2.0 beta 15 (32 bit). The IC_50 _values were calculated by the formula Y = 100*A1/(X + A1), where A1 = IC_50_, Y = response (Y = 100% when X = 0), X = inhibitory concentration. The IC_50 _values were compared by paired t tests. *p *< 0.05 was considered significant.

## Results

### Total antioxidant activity

**Figure 1:**
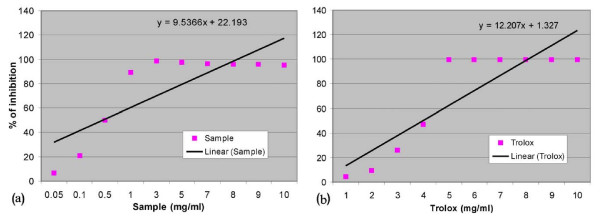
**Total antioxidant activity**. Total antioxidant activity of plant extract and trolox. Effect of (a) *Spondias pinnata *extract and (b) reference compound trolox on decolorization of ABTS radical cation. The percentage inhibition was plotted against the concentration of sample. All data are expressed as mean ± S.D. (n = 6).

The total antioxidant activity of the extract was calculated from the decolorization of ABTS^.+^, which was measured spectrophotometrically at 734 nm. Interaction with the extract or standard trolox suppressed the absorbance of the ABTS^.+ ^radical cation and the results, expressed as percentage inhibition of absorbance, are shown in figure 1(a) and figure 1(b), respectively. The TEAC value of the extract was 0.78 ± 0.02.

### Hydroxyl radical scavenging

**Figure 2:**
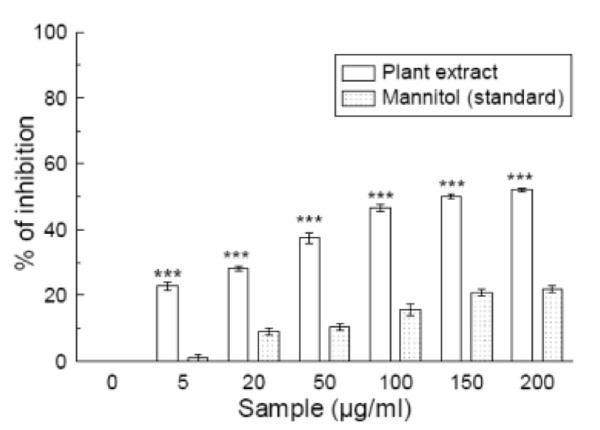
**Hydroxyl radical scavenging assay**. Hydroxyl radical scavenging activities of the *Spondias pinnata *extract and the reference compound mannitol. The data represent the percentage inhibition of deoxyribose degradation. The results are mean ± S.D. of six parallel measurements. ***p < 0.001 vs 0 μg/ml. IC_50 _= 112.18 ± 3.27 μg/ml. The IC_50 _value of the standard is 571.45 ± 20.12 μg/ml.

This assay shows the abilities of the extract and standard mannitol to inhibit hydroxyl radical-mediated deoxyribose degradation in an Fe^3+^-EDTA-ascorbic acid and H_2_O_2 _reaction mixture. The results are shown in figure 2. The IC_50 _values (Table 1) of the extract and standard in this assay were 112.18 ± 3.27 μg/ml and 571.45 ± 20.12 μg/ml, respectively. The IC_50 _value of the extract was less than that of the standard. At 200 μg/ml, the percentage inhibition values were 53.7% and 23% for *S. pinnata *and mannitol, respectively.

**Table 1:** Scavenging of reactive oxygen species and iron chelating activity (IC_50 _values) of *Spondias pinnata *and reference compounds

Activity	Extract/Reference	IC_50 _(#)
Hydroxyl radical (OH^.^) scavenging	*Spondias pinnata*	112.18 ± 3.27 (6)
	Mannitol	571.45 ± 20.12 (6)***
Superoxide anion (O_2 _^.-^) scavenging	*Spondias pinnata*	13.46 ± 0.66 (6)
	Quercetin	42.06 ± 1.35 (6)***
Nitric oxide radical (NO) scavenging	*Spondias pinnata*	24.48 ± 2.31 (6)
	Curcumin	90.82 ± 4.75 (6)***
Hydrogen peroxide (H_2_O_2_) scavenging	*Spondias pinnata*	44.74 ± 25.61 (6)
	Sodium pyruvate	3.24 ± 0.30 (6) *
Peroxynitrite (ONOO^-^) scavenging	*Spondias pinnata*	716.32 ± 32.25 (6)
	Gallic acid	876.24 ± 56.96 (6)***
Singlet oxygen (^1^O_2_) scavenging	*Spondias pinnata*	58.07 ± 5.36 (6)
	Lipoic acid	46.15 ± 1.16 (6) **
Hypochlorous acid (HOCl) scavenging	*Spondias pinnata*	127.99 ± 6.26 (6)
	Ascorbic acid	235.95 ± 5.75 (6)***
Iron Chelating	*Spondias pinnata*	66.54 ± 0.84 (6)
	EDTA	1.27 ± 0.05 (6)***

# Units of IC_50 _for all activities are μg/ml, except H_2_O_2 _scavenging, where the units are mg/ml. Data are expressed as mean ± S.D. Data in parenthesis indicate number of independent assays.

EDTA, Ethylenediamine tetraacetic acid.

* p < 0.05.

** p < 0.01.

*** p < 0.001 vs *Spondias pinnata*.

### Superoxide radical scavenging

**Figure 3:**
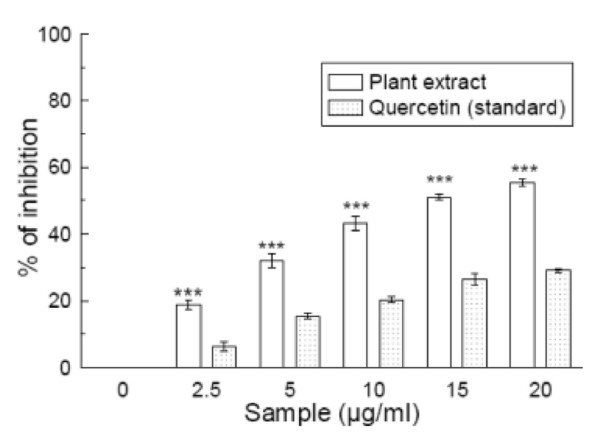
**Superoxide radical scavenging assay**. Scavenging effect of *Spondias pinnata *plant extract and the standard quercetin on superoxide radical. The data represent the percentage superoxide radical inhibition. All data are expressed as mean ± S.D. (n = 6). ***p < 0.001 vs 0 μg/ml. IC_50 _= 13.46 ± 0.66 μg/ml. The IC_50 _value of the standard is 42.06 ± 1.35 μg/ml.

The superoxide radicals generated from dissolved oxygen by PMS-NADH coupling can be measured by their ability to reduce NBT. The decrease in absorbance at 560 nm with the plant extract and the reference compound quercetin indicates their abilities to quench superoxide radicals in the reaction mixture. As shown in figure 3, the IC_50 _values (Table 1) of the plant extract and quercetin on superoxide scavenging activity were 13.46 ± 0.66 μg/ml and 42.06 ± 1.35 μg/ml, respectively. The IC_50 _value of the extract was less than that of the standard. At 20 μg/ml, the percentage inhibition of the plant extract was 55.2% whereas that of quercetin was 29.6%.

### Nitric oxide radical scavenging

**Figure 4:**
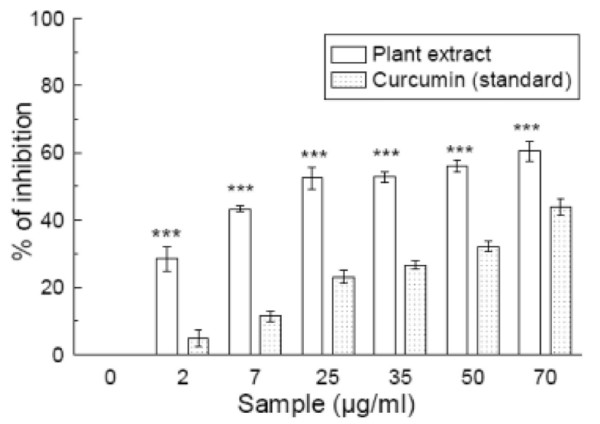
**Nitric oxide radical scavenging assay**. The nitric oxide radical scavenging activity of *Spondias pinnata *extract and the standard curcumin. The data represent the percentage nitric oxide inhibition. Each value represents mean ± S.D. (n = 6). ***p < 0.001 vs 0 μg/ml. IC_50 _= 24.48 ± 2.31 μg/ml. The IC_50 _value of the standard is 90.82 ± 4.75 μg/ml.

*S. pinnata *extract also caused a moderate dose-dependent inhibition of nitric oxide with an IC_50 _(Table 1) of 24.48 ± 2.31 μg/ml (figure 4). Curcumin was used as a reference compound and 90.82 ± 4.75 μg/ml curcumin was needed for 50% inhibition. The IC_50 _value of the extract was less than that of the standard. At 70 μg/ml, the percentage inhibition of the plant extract was 61.2% whereas that of curcumin was 44.1%.

### Hydrogen peroxide scavenging

**Figure 5:**
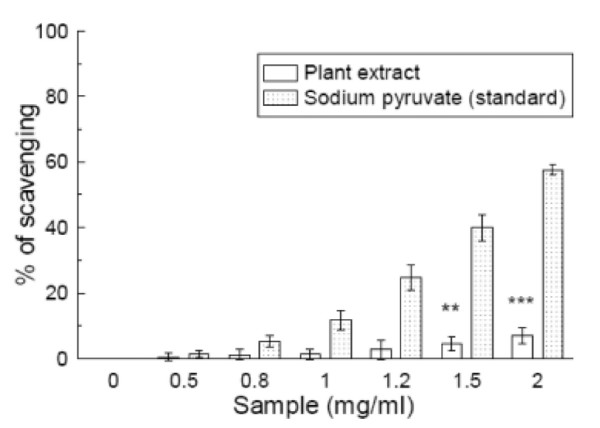
**H_2_O_2 _scavenging assay**. Effects of *Spondias pinnata *plant extract and the standard sodium pyruvate on the scavenging of H_2_O_2_. The data represent the percentage H_2_O_2 _scavenging. All data are expressed as mean ± S.D. (n = 6). **p < 0.01 and ***p < 0.001 vs 0 mg/ml. IC_50 _= 44.74 ± 25.61 mg/ml. The IC_50 _value of the standard is 3.24 ± 0.3 mg/ml.

Hydrogen peroxide scavenging was assayed by the FOX reagent method [14]. Figure 5 shows that the plant extract is a very poor scavenger of H_2_O_2 _(IC_50 _= 44.74 ± 25.61 mg/ml) compared to standard sodium pyruvate (IC_50 _= 3.24 ± 0.3 mg/ml). The IC_50 _value (Table 1) of the extract was greater than that of the standard. At a concentration of 2 mg/ml, the scavenging percentages were 6.5% and 57.7% for *S. pinnata *and sodium pyruvate, respectively.

### Peroxynitrite scavenging

**Figure 6:**
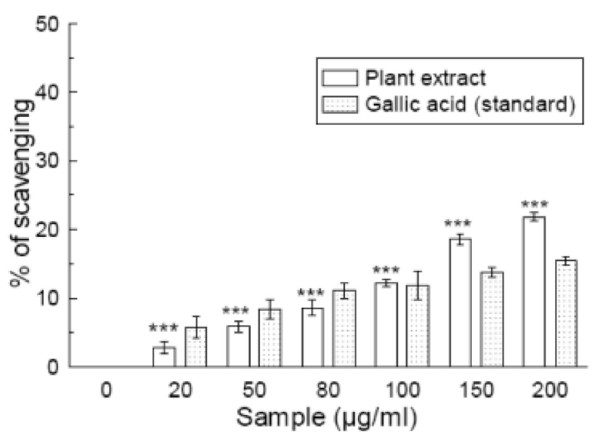
**Peroxynitrite anion scavenging assay**. The peroxynitrite anion scavenging activity of *Spondias pinnata *plant extract and the standard gallic acid. Each value represents mean ± S.D. (n = 6). ***p < 0.001 vs 0 μg/ml. IC_50 _= 716.32 ± 32.25 μg/ml. The IC_50 _value of the standard is 876.24 ± 56.96 μg/ml.

Figure 6 shows that the peroxynitrite scavenging activity of the plant extract was concentration-dependent. The calculated IC_50 _was 716.32 ± 32.25 μg/ml, which was lower than that of the reference compound gallic acid (IC_50 _= 876.24 ± 56.96 μg/ml) (Table 1), indicating that the sample is more potent scavenger of peroxynitrite than gallic acid. At 200 μg/ml, the scavenging percentages were 22.3% and 15.8% for *S. pinnata *and gallic acid, respectively.

### Singlet oxygen scavenging

**Figure 7:**
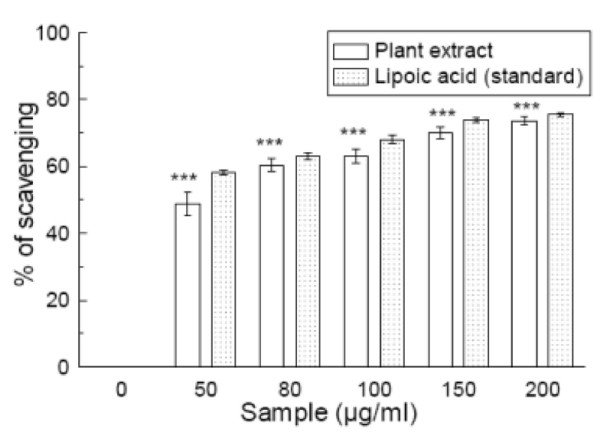
**Singlet oxygen scavenging assay**. Effects of *Spondias pinnata *plant extract and the standard lipoic acid on the scavenging of singlet oxygen. The results are mean ± S.D. of six parallel measurements. ***p < 0.001 vs 0 μg/ml. IC_50 _= 58.07 ± 5.36 μg/ml. The IC_50 _value of the standard is 46.15 ± 1.16 μg/ml.

*S. pinnata *extract was an effective scavenger of singlet oxygen (figure 7) and this activity was comparable to that of lipoic acid. The IC_50 _value (Table 1) of the test sample was 58.07 ± 5.36 μg/ml whereas that of lipoic acid was 46.15 ± 1.16 μg/ml. The IC_50 _value of the extract was higher than that of the reference compound. At 200 μg/ml, the percentage scavenging of the plant extract was 73.3% whereas that of lipoic acid was 75.3%.

### Hypochlorous acid scavenging

**Figure 8:**
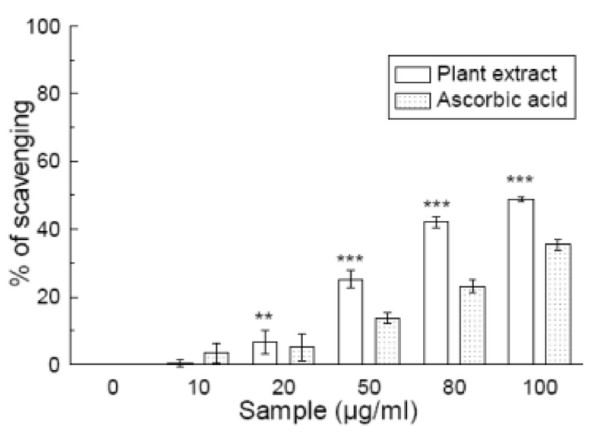
**HOCl scavenging assay**. Hypochlorous acid scavenging activities of *Spondias pinnata *plant extract and the standard ascorbic acid. All data are expressed as mean ± S.D. (n = 6). **p < 0.01 and ***p < 0.001 vs 0 μg/ml. IC_50 _= 127.99 ± 6.26 μg/ml. The IC_50 _value of the standard is 235.95 ± 5.75 μg/ml.

Figure 8 shows the dose-dependent hypochlorous acid scavenging activity of *S. pinnata *extract compared to that of ascorbic acid. The results indicate that the extract scavenged hypochlorous acid more efficiently (IC_50 _= 127.99 ± 6.26 μg/ml) than ascorbic acid (IC_50 _= 235.95 ± 5.75 μg/ml) (Table 1). At 100 μg/ml, the percentage scavenging of the plant extract was 49.2% whereas that of ascorbic acid was 34.6%.

### Fe^2+ ^chelation

**Figure 9:**
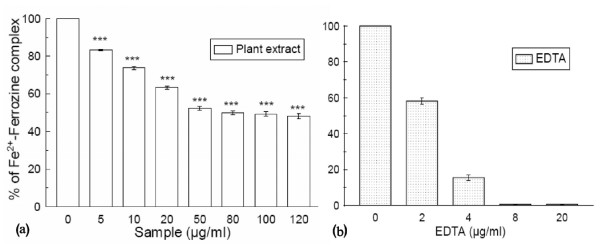
**Fe^2+ ^chelation assay**. Effects of (a) *Spondias pinnata *plant extract and (b) standard EDTA on ferrozine-Fe^2+ ^complex formation. The data are expressed as percentage inhibition of chromogen formation. The results are mean ± S.D. of six parallel measurements. ***p < 0.001 vs 0 μg/ml. IC_50 _= 66.54 ± 0.84 μg/ml. The IC_50 _value of the standard is 1.27 ± 0.05 μg/ml.

Ferrozine produces a violet complex with Fe^2+^. In the presence of a chelating agent, complex formation is interrupted and as a result the violet color of the complex is decreased. The results [figure 9(a) and figure 9(b)] demonstrated that formation of the ferrozine-Fe^2+ ^complex is inhibited in the presence of the test and reference compounds. The IC_50 _values (Table 1) of the plant extract and EDTA were 66.54 ± 0.84 μg/ml and 1.27 ± 0.05 μg/ml, respectively. At 120 μg/ml, the percentage inhibition of the plant extract was 51.8% whereas at 45 μg/ml that of EDTA was 99.5%.

### Reducing power

**Figure 10:**
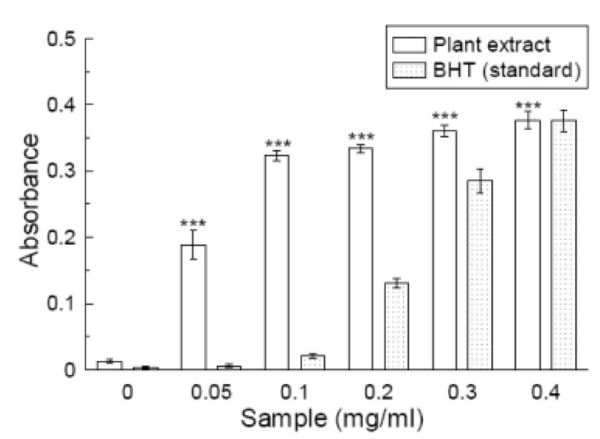
**Reducing power assay**. The reductive abilities of *Spondias pinnata *extract and the standard BHT. The absorbance (A_700_) was plotted against concentration of sample. Each value represents mean ± S.D. (n = 6). *** *p *< 0.001 vs 0 mg/ml.

As illustrated in figure 10, Fe^3+ ^was transformed to Fe^2+ ^in the presence of *S. pinnata *extract and the reference compound BHT to measure the reductive capability. At 0.1 mg/ml, the absorbances of the plant extract and BHT were 0.32 and 0.02, respectively, while at 0.4 mg/ml, the absorbances of both extract and BHT were almost the same. This result indicates that maximum activity is shown at a lower dose by the extract (0.1 mg/ml) than by BHT.

### Determination of total phenolic content

Phenolic compounds may contribute directly to antioxidative action. The total phenolic content was 91.47 ± 0.004 mg/ml gallic acid equivalent per 100 mg plant extract.

### Determination of total flavonoid content

The total flavonoid content of the 70% methanolic extract of S. pinnata was 350.5 ± 0.004 mg/ml quercetin equivalent per 100 mg plant extract.

## Discussion

In living systems, free radicals are constantly generated and they can cause extensive damage to tissues and biomolecules leading to various disease conditions, especially degenerative diseases, and extensive lysis [25]. Many synthetic drugs protect against oxidative damage but they have adverse side effects. An alternative solution to the problem is to consume natural antioxidants from food supplements and traditional medicines [26,27]. Recently, many natural antioxidants have been isolated from different plant materials [28,29]. The antioxidant and polyphenol contents of *S. pinnata *have also been determined by Chuyen et al. [30], using concentrated leaf extract. The antioxidant activity has been studied by the inhibition of ascorbic acid-induced lipid peroxidation and Catechin has been used as the standard for phenolic content, whereas in the present study, the antioxidant capacity of bark extract was measured by an improved ABTS radical cation decolorization assay and gallic acid was used as the standard for phenolic content measurement; both studies showed promising results. Therefore, it is clear that both the leaf and stem bark extracts of the plant have good antioxidant activities as well as high polyphenolic contents.

ABTS^.+ ^is a blue chromophore produced by the reaction between ABTS and potassium persulfate. Addition of the plant extract to this pre-formed radical cation reduced it to ABTS in a concentration-dependent manner. The results were compared with those obtained using trolox and the TEAC value demonstrates that the extract is a potent antioxidant.

Hydroxyl radicals are the major active oxygen species causing lipid peroxidation and enormous biological damage [31]. They were produced in this study by incubating ferric-EDTA with ascorbic acid and H_2_O_2 _at pH 7.4, and reacted with 2-deoxy-2-ribose to generate a malondialdehyde (MDA)-like product. This compound forms a pink chromogen upon heating with TBA at low pH [32]. When *S. pinnata *extract was added to the reaction mixture, it removed the hydroxyl radicals from the sugar and prevented the reaction. The IC_50 _value indicates that the plant extract is a better hydroxyl radical scavenger than the standard mannitol.

Superoxide anion is also very harmful to cellular components [33]. Robak and Glyglewski [34] reported that flavonoids are effective antioxidants mainly because they scavenge superoxide anions. As shown in figure 3, the superoxide radical scavenging activities of the plant extract and the reference compound are increased markedly with increasing concentrations. The results suggest that the plant extract is a more potent scavenger of superoxide radical than the standard quercetin.

It is well known that nitric oxide has an important role in various inflammatory processes. Sustained levels of production of this radical are directly toxic to tissues and contribute to the vascular collapse associated with septic shock, whereas chronic expression of nitric oxide radical is associated with various carcinomas and inflammatory conditions including juvenile diabetes, multiple sclerosis, arthritis and ulcerative colitis [35]. The toxicity of NO increases greatly when it reacts with superoxide radical, forming the highly reactive peroxynitrite anion (ONOO^-^) [36]. The nitric oxide generated from sodium nitroprusside reacts with oxygen to form nitrite. The extract inhibits nitrite formation by directly competing with oxygen in the reaction with nitric oxide. The present study proved that the extract studied has more potent nitric oxide scavenging activity than the standard curcumin.

Hydrogen peroxide is a weak oxidizing agent that inactivates a few enzymes directly, usually by oxidation of essential thiol (-SH) groups. It can cross cell membranes rapidly; once inside the cell, it can probably react with Fe^2+ ^and possibly Cu^2+ ^ions to form hydroxyl radicals and this may be the origin of many of its toxic effects [37]. From the results, it appeared that the H_2_O_2 _scavenging activity of the plant extract is negligible compared to that of the standard sodium pyruvate.

Peroxynitrite (ONOO^-^) is relatively stable compared to other free radicals but once protonated it forms the highly reactive peroxynitrous acid (ONOOH) [38]. Generation of excess ONOO^- ^leads to oxidative damage and tissue injury [39]. Peroxynitrite bleaches Evans Blue by oxidizing it. According to the present results, the plant extract inhibits Evans Blue bleaching by scavenging peroxynitrite and its activity is greater than that of the reference gallic acid.

Singlet oxygen is generated in the skin by ultraviolet radiation. It is a high energy form of oxygen and is known as one of the ROS. Singlet oxygen induces hyperoxidation and oxygen cytotxicity and decreases antioxidative activity [40]. The present study indicates that the *S. pinnata *extract has good scavenging activity for singlet oxygen but is not as efficient as the standard lipoic acid.

At sites of inflammation, the oxidation of Cl^- ^ions by the neutrophil enzyme myeloperoxidase results in the production of another harmful ROS, hypochlorous acid [41]. HOCl has the ability to inactivate the antioxidant enzyme catalase through breakdown of the heme prosthetic group. Catalase inactivation is inhibited in the presence of the extract, signifying its HOCl scavenging activity. The results suggest that *S. pinnata *is a more efficient scavenger than the standard ascorbic acid.

Iron can stimulate lipid peroxidation by the Fenton reaction (H_2_O_2 _+ Fe^2+ ^= Fe^3+ ^+ OH^- ^+ OH^.^) and can also accelerate lipid peroxidation by decomposing lipid hydroperoxides into peroxyl and alkoxyl radicals that can perpetuate the chain reaction [42]. Metal chelating capacity is significant since it reduces the concentration of the transition metal that catalyzes lipid peroxidation [43]. According to the results, the plant extract is not as good as the standard EDTA; but the decrease in concentration-dependent color formation in the presence of the extract indicates that it has iron chelating activity.

The reducing capacity of a compound may serve as a significant indicator of its potential antioxidant activity. However, the activities of antioxidants have been attributed to various mechanisms such as prevention of chain initiation, decomposition of peroxides, reducing capacity and radical scavenging [44]. As shown in figure 7, the reducing power of the plant extract was compared with the standard BHT and found to be superior.

The results indicate that *S. pinnata *plant extract contains significant amounts of flavonoids and phenolic compounds. Both these classes of compounds have good antioxidant potential and their effects on human nutrition and health are considerable. The mechanism of action of flavonoids is through scavenging or chelation [45]. Phenolic compounds are also very important plant constituents because their hydroxyl groups confer scavenging ability [44].

## Conclusion

On the basis of the results obtained in the present study, it is concluded that a 70% methanolic extract of *Spondias pinnata *bark, which contains large amounts of flavonoids and phenolic compounds, exhibits high antioxidant and free radical scavenging activities. It also chelates iron and has reducing power. These *in vitro *assays indicate that this plant extract is a significant source of natural antioxidant, which might be helpful in preventing the progress of various oxidative stresses. However, the components responsible for the antioxidative activity are currently unclear. Therefore, further investigation is needed to isolate and identify the antioxidant compounds present in the plant extract. Furthermore, the *in vivo *antioxidant activity of this extract needs to be assessed prior to clinical use.

## Competing interests

The authors declare that they have no competing interests.

## Authors' contributions

BH: Performed the study.

SB: Design, analysis and acquisition of data.

NM: Supervised the study design and drafted the manuscript.

## Pre-publication history

The pre-publication history for this paper can be accessed here:



## References

[B1] Braca A, Sortino C, Politi M, Morelli I, Mendez J (2002). Antioxidant activity of flavonoids from *Licania licaniaeflora*. J Ethnopharmacol.

[B2] Maxwell SR (1995). Prospects for the use of antioxidant therapies. Drugs.

[B3] Niki E, Shimaski H, Mino M (1994). Antioxidantism-free radical and biological defense. Gakkai Syuppn Center, Tokyo.

[B4] Tandon S, Rastogi RP (1976). Studies on the chemical constituents of *Spondias pinnata*. Planta Med.

[B5] Ghosal PK, Thakur S (1981). Structural features of the acidic polysaccharide of *Spondias pinnata *gum exudates. Carbohydr Res.

[B6] Bibitha B, Jisha VK, Salitha CV, Mohan S, Valsa AK (2002). Antibacterial activity of different plant extracts. Indian J Microbiol.

[B7] Mahanta RK, Rout SD, Sahu HK (2006). Ethnomedicinal plant resources of Similipal biosphere reserve, Orissa, India. Zoos Print J.

[B8] Leksomboon C, Thaveechai N, Kositratana W (2001). Potential of plant extracts for controlling citrus canker of lime. Kasetsart J (Nat Sci).

[B9] Re R, Pellegrini N, Proteggente A, Pannala A, Yang M, Rice-Evans C (1999). Antioxidant activity applying an improved ABTS radical cation decolorization asszy. Free Rad Biol Med.

[B10] Elizabeth K, Rao MNA (1990). Oxygen radical scavenging activity of curcumin. Int J Pharmaceut.

[B11] Fontana M, Mosca L, Rosei MA (2001). Interaction of enkephalines with oxyradicals. Biochem Pharmacol.

[B12] Garratt DC (1964). The quantitative analysis of Drugs.

[B13] Long LH, Evans PJ, Halliwell B (1999). Hydrogen peroxide in human urine: implications for antioxidant defense and redox regulation. Biochem Biophys Res Commun.

[B14] Floriano-Sánchez E, Villanueva C, Medina-Campos ON, Rocha D, Sánchez-González DJ, Cárdenas-Rodríguez N, Pedraza-Chaverrí J (2006). Nordihydroguaiaretic acid is a potent in vitro scavenger of peroxynitrite, singlet oxygen, hydroxyl radical, superoxide anion, andhypochlorous acid and prevents in vivo tyrosine nitration in lung. Free Radic Res.

[B15] Beckman JS, Chen H, Ischiropulos H, Crow JP (1994). Oxidative chemistry of peroxynitrite. Methods Enzymol.

[B16] Bailly F, Zoete V, Vamecq J, Catteu JP, Bernier JL (2000). Antioxidant actions of ovothiol-derived 4-mercaptoimidazoles: glutathione peroxidase activity and protection against peroxynitrite-induced damage. FEBS Lett.

[B17] Chakraborty N, Tripathy BC (1992). Involvement of singlet oxygen in 5-aminolevulinic acid-induced photodynamic damage of cucumber (*Cucumbis sativus *L.) chloroplasts. Plant Physiol.

[B18] Pedraza-Chaverrí J, Barrera D, Maldonado PD, Chirino YI, Macías-Ruvalcaba NA, Medina-Campos ON, Castro L, Salcedo MI, Hernández-Pando R (2004). S-allylmercaptocysteine scavenges hydroxyl radical and singlet oxygen in vitro and attenuates gentamicininduced oxidative and nitrosative stress and renal damage *in vivo*. BMC Clin Pharmacol.

[B19] Aruoma OI, Halliwell B (1987). Action of hypochlorous acid on the antioxidant protective enzymes superoxide dismutase, catalase and glutathione peroxidase. Biochem J.

[B20] Pedraza-Chaverrí J, Arriaga-Noblecía G, Medina-Campos ON (2007). Hypochlorous acid scavenging capacity of garlic. Phytother Res.

[B21] Haro-Vicente JF, Martinez-Gracia C, Ros G (2006). Optimization of *in vitro *measurement of available iron from different fortificants in citric fruit juices. Food Chem.

[B22] Oyaizu M (1986). Studies on products of browning reactions: antioxidant activities of products of browning reaction prepared from glucose amine. Jap J Nutr.

[B23] Singleton VL, Rossi JA (1965). Colorimetry of total phenolics with phosphomolybdic-phosphotungstic acid reagents. Am J Enol Vitic.

[B24] Zhishen J, Mengcheng T, Jianming W (1999). The determination of flavonoid content in mulberry and their scavenging effects on superoxide radicals. Food Chem.

[B25] Halliwell B, Gutteridge JM (1998). Free radicals in biology and medicine.

[B26] Yazdanparast R, Ardestani A (2007). *In vitro *antioxidant and free radical scavenging activity of *Cyperus rotundus*. J Med Food.

[B27] Yazdanparast R, Bahramikias S, Ardestani A (2008). *Nasturtium oficinale *reduces oxidative stress and enhances antioxidant capacity in hypercholesterolaemic rats. Chem Biol Interact.

[B28] Packer L, Ong ASH (1997). Biological oxidants and antioxidants: Molecular mechanisms and health effects.

[B29] Jovanovic SV, Simic MG (2000). Antioxidants in nutrition. Ann NY Acad Sci.

[B30] Mai TT, Thu NN, Tien PG, Chuyen NV (2007). Alpha-Glucosidase inhibitory and antioxidant activities of Vietnamese edible plants and their relationships with polyphenol contents. J Nutr Sci Vitaminol.

[B31] Aurand LW, Boone NH, Giddings GG (1977). Superoxide and singlet oxygen in milk lipid peroxidation. J Dairy Sci.

[B32] Halliwell B, Gutteridge JMC, Aruoma OI (1987). The deoxyribose method: a simple 'test tube' assay for determination of rate constants for reaction of hydroxyl radicals. Anal Biochem.

[B33] Korycka-Dahl M, Richardson T (1978). Photogeneration of superoxide anion in serum of bovine milk and in model systems containing riboflavin and amino acids. J Dairy Sci.

[B34] Robak J, Gryglewski IR (1988). Flavonoids are scavengers of superoxide anions. Biochem Pharmacol.

[B35] Tylor BS, Kion YM, Wang QI, Sharpio RA, Billiar TR, Geller DA (1997). Nitric oxide down regulates hepatocyte-inducible nitric oxide synthase gene expression. Arch Surg.

[B36] Huie RE, Padmaja S (1993). The reaction of NO with superoxide. Free Radic Res Commun.

[B37] Miller MJ, Sadowska-Krowicka H, Chotinaruemol S, Kakkis JL, Clark DA (1993). Amelioration of chronic ileitis by nitric oxide synthase inhibition. J Pharmacol Exp Ther.

[B38] Balavoine GG, Geletti YV (1999). Peroxynitrite scavenging by different antioxidants. Part 1: convenient study. Nitric oxide.

[B39] Ischiropoulos H, al-Mehdi AB, Fisher AB (1995). Reactive species in ischemic rat lung injury: contribution of peroxynitrite. Am J Physiol.

[B40] Kochevar EI, Redmond WR (2000). Photosnsitized production of singlet oxygen. Methods Enzymol.

[B41] Aruoma OI, Halliwell B, Hoey BM, Butler J (1989). The antioxidant action of N-acetylcysteine: Its reaction with hydrogen peroxide, hydroxyl radical, superoxide, and hypochlorous acid. Free Rad Biol Med.

[B42] Halliwell B (1991). Reactive oxygen species in living systems: source, biochemistry, and role in human disease. Am J Med.

[B43] Duh PD, Tu YY, Yen GC (1999). Antioxidant activity of water extract of Harng Jyur (*Chrysenthemum morifolium *Ramat). Lebnes wiss Technol.

[B44] Yildirim A, Mavi A, Oktay M, Kara AA, Algur OF, Bilaloglu V (2000). Comparison of antioxidant and antimicrobial activities of Tilia (*Tilia argentea *Desf Ex DC), Sage (*Savia triloba *L.), and Black Tea (*Camellia sinensis*) extracts. J Agric Food Chem.

[B45] Cook NC, Samman S (1996). Flavonoids-chemistry, metabolism, cardioprotective effects, and dietary sources. J Nutr Biochem.

